# Independent Component Analysis for Unraveling the Complexity of Cancer Omics Datasets

**DOI:** 10.3390/ijms20184414

**Published:** 2019-09-07

**Authors:** Nicolas Sompairac, Petr V. Nazarov, Urszula Czerwinska, Laura Cantini, Anne Biton, Askhat Molkenov, Zhaxybay Zhumadilov, Emmanuel Barillot, Francois Radvanyi, Alexander Gorban, Ulykbek Kairov, Andrei Zinovyev

**Affiliations:** 1 Institut Curie, PSL Research University, 75005 Paris, France (N.S.) (U.C.) (E.B.) (F.R.); 2 INSERM U900, 75248 Paris, France; 3 CBIO-Centre for Computational Biology, Mines ParisTech, PSL Research University, 75006 Paris, France; 4 Centre de Recherches Interdisciplinaires, Université Paris Descartes, 75004 Paris, France; 5 Multiomics Data Science Research Group, Quantitative Biology Unit, Luxembourg Institute of Health (LIH), L-1445 Strassen, Luxembourg; 6 Computational Systems Biology Team, Institut de Biologie de l’Ecole Normale Supérieure, CNRS UMR8197, INSERM U1024, Ecole Normale Supérieure, PSL Research University, 75005 Paris, France; 7 Centre de Bioinformatique, Biostatistique et Biologie Intégrative (C3BI, USR 3756 Institut Pasteur et CNRS), 75015 Paris, France; 8 Laboratory of Bioinformatics and Systems Biology, Center for Life Sciences, National Laboratory Astana, Nazarbayev University, 010000 Nur-Sultan, Kazakhstan (A.M.) (Z.Z.) (U.K.); 9 University Medical Center, Nazarbayev University, 010000 Nur-Sultan, Kazakhstan; 10 CNRS, UMR 144, 75248 Paris, France; 11 Center for Mathematical Modeling, University of Leicester, Leicester LE1 7RH, UK; 12 Lobachevsky University, 603022 Nizhny Novgorod, Russia

**Keywords:** independent component analysis, cancer, omics data, dimension reduction, data analysis, data integration

## Abstract

Independent component analysis (ICA) is a matrix factorization approach where the signals captured by each individual matrix factors are optimized to become as mutually independent as possible. Initially suggested for solving source blind separation problems in various fields, ICA was shown to be successful in analyzing functional magnetic resonance imaging (fMRI) and other types of biomedical data. In the last twenty years, ICA became a part of the standard machine learning toolbox, together with other matrix factorization methods such as principal component analysis (PCA) and non-negative matrix factorization (NMF). Here, we review a number of recent works where ICA was shown to be a useful tool for unraveling the complexity of cancer biology from the analysis of different types of omics data, mainly collected for tumoral samples. Such works highlight the use of ICA in dimensionality reduction, deconvolution, data pre-processing, meta-analysis, and others applied to different data types (transcriptome, methylome, proteome, single-cell data). We particularly focus on the technical aspects of ICA application in omics studies such as using different protocols, determining the optimal number of components, assessing and improving reproducibility of the ICA results, and comparison with other popular matrix factorization techniques. We discuss the emerging ICA applications to the integrative analysis of multi-level omics datasets and introduce a conceptual view on ICA as a tool for defining functional subsystems of a complex biological system and their interactions under various conditions. Our review is accompanied by a Jupyter notebook which illustrates the discussed concepts and provides a practical tool for applying ICA to the analysis of cancer omics datasets.

## 1. Introduction

Cancer research is one of the most important providers of large-scale molecular profiling data, which help in understanding not only the state of human cells in disease but also shed light on the normal physiological processes measurable and detectable in various kinds of omics datasets. Determining robust and biologically meaningful ways of quantifying cellular and organismal and normal and pathological physiology using high-throughput molecular data remains a major challenge (making biology a quantitative science). Different kinds of biological processes leave characteristic traces at different levels of genome-wide measurements depending on their nature and timescales: some significantly affect transcriptomes, some rather modify DNA methylation programs or mutational spectrum, others are measurable only at the level of proteome and phosphoproteome. In order to reliably quantify some of these biological mechanisms, one will need to design multi-omics signatures spanning several levels of molecular data descriptions. On top of this, various technical factors interplay with biological ones, frequently in a way which makes it difficult to clearly distinguish both.

Rarely does molecular data “speak for themselves”: they need to be properly pre-processed, analyzed in the light of mathematical modeling, statistical assumptions, and prior biological knowledge and, finally, should be represented at some pre-defined level of abstraction. In this sense, one of the simplest paradigms of *linear mixture of signals* plays a pivotal role in the modern molecular data analysis. In this framework, one assumes that a measurable elementary quantity such as expression of a single gene is a result of weighted summation of some latent, and not always directly observable, factor activities which should have associated numerical values. The nature, the number of factors and the way they are represented numerically can be known or unknown in advance. A toolbox of existing mathematical approaches provides concrete scenarios in which the additive factors can be determined and quantified, under acceptance of certain assumptions about the statistical properties of their numerical values or the weights connecting them to the measurements.

**Figure 1:**
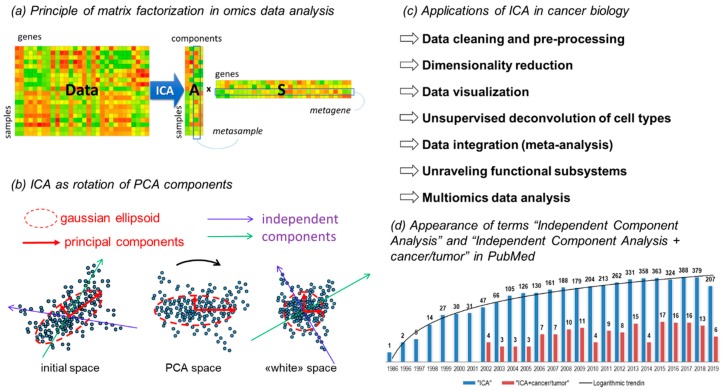
Independent component analysis (ICA) is a standard tool for reducing the complexity of omics datasets in cancer biology. (**a**) ICA belongs to the family of matrix factorization methods, approximating a 2D matrix by a product of two much smaller matrices, containing metagenes and metasamples, in the case of omics data. (**b**) ICA can be considered as a rotation of PCA axes, after data “whitening” (i.e., orienting the Gaussian ellipsoid along the coordinate axes and scaling them to unit variance). (**c**) The major types of applications of ICA in cancer biology. (**d**) The number of publications in PubMed mentioning ICA and the number of publications simultaneously mentioning ICA and “tumor” or “cancer”.

One of the standard methods in such a toolbox is independent component analysis (ICA) having a long standing history of application to biological data, including the analysis of molecular profiles (mainly, transcriptomic). Formally, ICA belongs to a family of methods called matrix factorizations (Figure 1), the most popular other representatives of which are principal component analysis (PCA) or the very similar singular value decomposition (SVD), and non-negative matrix factorization (NMF).

The first applications of ICA in biology contrasted it to PCA and standard clustering methods and found that the factors determined through ICA are easier to interpret biologically [1,2]. This raised an increase in interest of ICA and its applications in various contexts, and, in particular, in cancer biology [3,4]. The success of ICA can be connected to the nature of the statistical assumptions which are used to define the method, that match well the underlying high-dimensional distributions of omics datasets. The principles of ICA are briefly introduced in Section 2.1.

Independent component analysis and matrix factorization approaches are standard methods in the rapidly growing arsenal of machine learning methods applied to the molecular biology and medical data. At the same time, remarkable success has recently been achieved in applying deep learning techniques in certain fields of cancer biology such as clinical imaging of various kinds [5,6,7,8,9]. Deep learning has been successfully used in automating the diagnosis and prognosis of several cancer types, claiming to be competitive with human pathologists [10,11]. Successful applications of deep learning methods to multi-omics data have been recently reported, such as in Reference [12]. One should also notice that there exists a certain level of controversy in assessing the actual success of this rapidly growing area [13] and an important methodological discussion on the “deep” versus “shallow” methods in real applications [14]. Reviewing any statistical method today should necessarily take into account the existing intrinsic competition between this relatively recent trend and more “classical” areas of machine learning, even though many of them, including ICA, are rooted in the artificial neural network theory [15].

Over the last decade, significant experience in applying ICA to different kinds of omics data for addressing various problems has been obtained, including data pre-processing, task of cell type deconvolution, and meta-analysis of multiple omics datasets (Figure 1c). In this paper, we reviewed most of the recent achievements in computational cancer biology research where ICA was used as the main data analysis tool. We also discussed the practices of ICA applications which appeared to be successful in various contexts.

This review is accompanied by interactive Jupyter notebook located at https://github.com/sysbio-curie/ICA-in-Cancer-research-review-materials.

## 2. Methodology of ICA Application to Cancer Omics Data

### 2.1. Brief Introduction into Matrix Factorization Applied to Omics Data

Independent component analysis belongs to a family of matrix factorization methods. Each of these methods takes a rectangular matrix $X\in {\mathbb{R}}_{m}^{N}$ of measurements (in sufficiently a large number of observed samples, *N*, and with number of observed features, *m*) as an input and approximates it as a sum of products of *p* pairs of vectors of size *N* and *m*. The fundamental equation for all matrix factorization methods states (note that the product of ***a_k_*** and ***s_k_*** vectors gives a one-rank matrix of the same dimension as ***X***):

(1) $$X\approx \sum_{k=1}^{p}a_{k}\times s_{k}\;(*)$$

and the problem of matrix factorization is to find a set of ***a_k_*** and ***s_k_*** such that:

(2) $$\left\|X-\sum_{k=1}^{p}a_{k}\times s_{k}\|{}^{2}\to min\;(**\right)$$

where ‖..‖ is a suitable matrix norm which is most frequently the sum of the Euclidean norms of the columns of the matrix.

Each vector pair ***a_k_*** and ***s_k_*** will be called a component throughout this review. Therefore, a component is represented by a vector ***s_k_*** of size *m* containing weights of omics variables (genes, proteins, CpG sites, etc.). At the same time a component is associated to a vector ***a_k_*** of size *N*, containing contributions of the component to measured samples. We will use these notations and meaning of ***a_k_*** and ***s_k_*** vectors throughout the whole review.

In the matrix factorization literature, various terms are used to denote the elements of the vectors ***a_k_*** and ***s_k_***. For example, the terms “loadings”, “activations”, “factor strength” or “sample-associated weights” have been used to denote the elements of ***a_k_*** vectors. The matrix composed from the ***a_k_*** vectors is sometimes called the “mixing matrix” and denoted as ***A***. The elements of ***s_k_*** vectors have been called “weights of the component” or “signals” and the matrix composed of them (denoted as ***S***) is sometimes called the “signal matrix”. Moreover, ***s_k_*** vectors themselves are frequently referred to as “components” or “factors”. 

In the context of transcriptomic data analysis, the **s_k_** vector is frequently named a metagene [16]; in the case of other data types one can use similar naming, e.g., a metaCpG for the analysis of DNA methylation profiles. Further we will use the term metagene (or metagene weights for the individual elements) to refer to vector **s_k_** even when describing application of ICA to various data types. Similarly, the **a_k_** vectors are sometimes called metasamples, and we will adopt this term in the text (referring to the individual vector elements as metasample weights), see Figure 1a.

Intuitively, a transcriptome of a biological sample is described as a combined action of *p* metagenes. Each metagene abstractly represents a molecular program (called a functional subsystem further in the text) by assigning a numerical weight to each gene of the organismal genome. The activity of metagenes in a sample is combined additively, and each metagene acts on a sample with a sample-specific strength or activity. Activities of the same metagene over all measured samples is called a metasample. A metasample is the profile of the corresponding metagene activity similarly to a gene expression profile across samples.

In the equation (*), only the ***X*** matrix is known; the ***a_k_*** and ***s_k_*** vectors are unknown. As such, the problem of matrix factorization (**) is heavily underdetermined, and additional constraints need to be introduced on ***a_k_*** and ***s_k_*** vectors in order to find its solution. First of all, it can be required that the all ***a_k_*** vectors would have length one. 

Furthermore, one can require orthogonality of the ***a_k_*** vectors: (***a_i_***,***a_j_***) = 0, for *i* ≠ *j* and that the solution of (**) should give the same result for different orders of matrix decomposition *p*, i.e., ***a_k_*** and ***s_k_*** vectors computed for the order *p* = *p*’ would be the same as for the decomposition of order *p*’’ > *p*’. In this case, solving (**) is equivalent to computing the singular value decomposition (SVD) of ***X*** and gives a set of principal components. There exist several ways to introduce PCA, as reviewed in Reference [17].

Alternatively, one can require that all elements of ***a_k_*** and ***s_k_*** vectors would be non-negative. This constrains the problem (**) and leads to NMF. The simplest approach to solve (**) with these constraints is to repetitively apply the non-negative least squares regression method, considering ***a_k_*** as unknown at one iteration and ***s_k_*** as unknown at the next iteration, until convergence to a local minimum.

When computing ICA, the resulting components are required to be *as mutually independent as possible*. More precisely, the elements of vectors ***s_k_*** (or sometimes, vectors ***a_k_***) have to represent maximally mutually independent distributions, for different *k*. The perfect independence would mean that the joint probability distribution *P*(*s_1_*,…,*s_p_*) can be factorized as $P\left(s_{1},\ldots ,s_{p}\right)\;=\;P_{1}\left(s_{1}\right)\times \;P_{2}\left(s_{2}\right)\ldots \times \;P_{p}\left(s_{p}\right)$. Here, one assumes that the elements of vectors ***s_k_*** are i.i.d. samples of the underlying probability distributions *P_k_*(*s_k_*).

**Figure 2:**
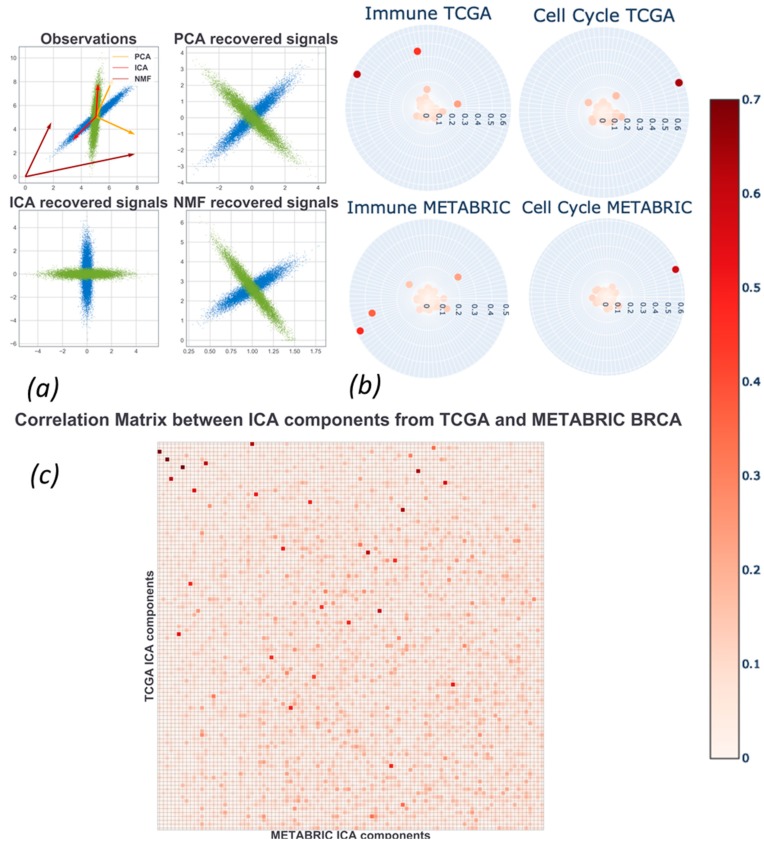
Features of ICA applied to a synthetic (**a**) and two real-life datasets (breast cancer The Cancer Genome Atlas (TCGA) and Molecular Taxonomy of Breast Cancer International Consortium (METABRIC) transcriptomic datasets) (**b**,**c**). (**a**) Independent Component Analysis is able to disentangle (or deconvolute) two intersecting Gaussian distributions with coinciding means and whose principal axes form a sharp angle; (**b**) 100 order ICA decomposition of the TCGA and METABRIC datasets. Each component represented as a metagene was correlated to either immune infiltration-related or proliferation-related meta-metagenes derived from Reference [33]. This analysis shows that only one of the components was strongly correlated to the cell-cycle, while several can be associated with the presence of an immune-infiltrated ICA-derived signature (this, probably, signifies the ability of ICA to deconvolute the major immune cell types in an unsupervised manner (see, Reference [42]); (**c**) correlations matrix between the metagenes of independent components extracted from the TCGA and METABRIC separately. It shows that, for some components computed for different datasets, there exists a strong and unique association between them, indicating the high reproducibility of the ICA results (e.g., see Reference [38]).

From the different nature of the constraints follow different properties of matrix factorization algorithms (see Reference [18] and Figure 2a). The PCA solves a convex quadratic optimization problem, which has a unique global minimum. The principal components are orthogonal and can be naturally ranked by the amount of explained variance. The NMF and ICA problems are not convex; therefore, the algorithms used to solve the optimization problem provide solutions depending on the component initialization. By construction, NMF and ICA do not lead to an orthogonal set of ***a_k_*** vectors and the components cannot be naturally ranked. The NMF components contain only non-negative elements, which makes the intuitive picture of the additive action of metagenes simpler to interpret, while in PCA and ICA some metagenes can cancel the action of other metagenes if they are summed up with different signs.

### 2.2. ICA Algorithms 

One of the historically first and still popular practical algorithms for solving ICA problem is based on the general Infomax (or maximum entropy) principle [19]. Indeed, the problem of ICA consists in minimizing the mutual information among individual components (represented by finite ***s_k_*** vectors). It can be shown that maximizing entropy of joint distributions of pairs of ***s_k_*** leads to minimizing their mutual information. 

It appeared also that under some assumptions, minimizing the mutual information is equivalent to maximizing the non-Gaussianity of the individual ***s_k_*** distributions [20]. Quantification of non-Gaussianity for continuous distributions involves negentropy (or Gibbs free energy, in physics). Negentropy measures the departure from Gaussianity of a random vector of density P(u) by comparing its entropy to the entropy of a normal distribution with same mean and variance. The entropy is defined with a negative sign (S=−∫P(u)logP(u)du) and the negentropy is, therefore, a non-negative function reaching zero only for the standardized normal distribution. For the mathematical details, we refer the reader to the classical works [19,20]. 

Since the length of the ***s_k_*** or ***a_k_*** vectors is always finite in real-life applications, one needs to introduce the way to effectively approximate it from the finite samples. For this purpose, various surrogate functions (called non-linearity functions) have been proposed, one the most popular of which involving the kurtosis. Empirically, kurtosis was found to be an appropriate choice of non-linearity in the analysis of transcriptomic data. Other types of non-linearity functions have been suggested; however, the appropriate choice of non-linearity for applying ICA to different kinds of omics measurements remains an open question. The two most popular ICA algorithms based on non-Gaussianity maximization are fastICA [20] and joint approximation diagonalization of Eigen-matrices (JADE) [21]. Most of the recent applications of ICA to omics data were based on fastICA, utilizing approximate Newton iterations to optimize a non-Gaussianity measure. However, other approaches to computing independent components have been used such as the product density estimation-based method (ProDenICA), claimed to have higher sensitivity to a wider range of source distributions than fastICA [22,23].

A typical preprocessing step used before application of ICA algorithms is the so-called data whitening or sphering (see Figure 1b). Whitening imposes unit variance along each axis. It consists of choosing a number of significant principal components, thus defining the resulting number of factors and then rotating the data to the basis of principal Gaussian ellipsoid axes and scaling along the principal axes to the unit variance. In the geometrical language, the Mahalanobis metrics are introduced into the data space instead of the usual Euclidean. Therefore, after whitening, the covariance matrix of the reduced dataset becomes the identity matrix and PCA becomes inapplicable, since all Gaussian signals have been erased from the data. This makes the use of higher-order moments for finding a rotation of the orthonormal coordinate basis easier, which would maximize the non-Gaussianity of the data point projection distributions along each axis. After such a rotation, in the whitened space, the vectors corresponding to the new axes remain orthogonal while in the original data space they can be strongly correlated (see Figure 2a). Because of the use of whitening as a preprocessing procedure, ICA is frequently considered as a step on top of PCA, consisting in rotating the coordinate system, by exploiting the information contained in higher than second moments of the multivariate data distribution (Figure 1c).

Various flavors of ICA have been suggested and some of them were tried on omics data. Bayesian ICA with prior constraints have been suggested and tried on the metabolomics data [24]. The prior constraints can be non-negativity of the ***a_k_*** and ***s_k_*** vector elements. This allows combining the nice properties of non-negative mixture problem and the requirement for mutual independence of the components. A kernel version of ICA have been developed [25] and sparse ICA was proposed in Reference [26], but both have not yet found wide applications in omics data analysis (though kernel ICA was exploited in Reference [27]). Finally, tensorial ICA was recently developed in References [28,29] and recently applied to the joint analysis of gene expression, copy number changes, and DNA methylation data from colon cancer with some promising results (see more in Section 3.5).

Some flavors of ICA seems interesting to explore more in biological applications, in the view of the concept of the integration of functional subsystems (see Section 3.6), such as tree-dependent component analysis (TCA) [30]. This variant of ICA allows clustering of the components such that they remain independent between the clusters and dependent within them. It was tested on fMRI data [31], but not yet on large-scale omics datasets.

### 2.3. Various Ways to Apply ICA to Omics Data

Besides the choice of ICA algorithm (which is frequently fastICA), there are several choices to be made when ICA is applied to omics datasets. 

The first evident but non-trivial choice concerns a necessity for data log-transformation, which is especially important in the case of gene expression and protein expression data. On one hand, it is strongly desirable in the case of, for example, RNA-Seq data, since empirically they are found to be characterized by log-normal distribution. When ICA is applied to non-transformed data, the resulting components are frequently dominated by single genes or single samples (e.g., each sample acts as an independent component), which contradicts the initial concept of linear mixture (nothing or almost nothing is mixed in this case). Simple log-transformation usually fixes this issue. However, log-transformation makes the direct interpretation of the ICA model difficult, since, formally speaking, one deals with a multiplicative rather than an additive model of signal mixture. This is particularly important for the applications of ICA in the field of cell type deconvolution where the linearity assumption is explicitly made for mixing transcriptomes of different cell types (see Reference [32] which cites a number of references studying the issue of data log transformation). Another aspect is that log-transformation can amplify small values, sometimes creating a heavy tail of negative values, characterized by strong non-Gaussianity and affecting the ICA determination. In practice, log-transformation can be recommended after adding a small value (e.g., 1 sequence count) to all data matrix entries, before taking the log. This is especially true in the case of sparse single cell RNA-Seq data, where the majority of matrix entries can equal to zero. On the other hand, choosing a threshold for small expression values looks like an arbitrary choice, especially if the RNA-Seq data have been normalized beforehand. Despite these difficulties, in most of the applications of ICA to RNA-Seq data analysis, the so called “log(x+1)” transformation can be advised: empirically, it is found to lead to more stable and biologically interpretable components. The problem of log transformation became more relevant after introducing sequencing technologies such as RNA-Seq; for microarray-based methods, the gene expression measurements were frequently provided in log scale, after some standard normalizations such as robust multichip average.

Another choice in applying ICA to a matrix of omics measurements is the choice between what distribution independence (or non-Gaussianity) is maximized [18]. One can maximize the independence of metagenes (vectors ***s_k_***) or metasamples (vectors ***a_k_***). Technically, the first case corresponds to the application of ICA algorithm to the initial matrix ***X*** containing samples as rows and omics variables as columns, and the second case corresponds to the application of ICA to the transposed matrix ***X***. Surprisingly, both ways of applying ICA to omics data are wide-spread, and sometimes it makes an effort to figure out in which way ICA was applied. Some studies aim at maximizing the non-Gaussianity of metagenes [2,33,34,35], while others maximize non-Gaussianity of metasamples [36,37]. Empirically it was shown that maximizing the non-Gaussianity of metagenes is clearly preferable in gene expression analyses to maximizing the non-Gaussianity of metasamples [38]. This choice leads to much better reproducibility of metagenes in independent datasets as well as to better interpretability of the components computed within the same dataset.

Furthermore, in several studies it was found that stabilized or consensus independent components have better characteristics in terms of generalization and interpretation [34,38,39,40,41]. By stabilization one usually means re-computing ICA using multiple random initialization with subsequent clustering of the resulting components [40,41]. Alternatively, stabilization can be performed through sub-sampling, i.e., computing ICA multiple times after removing a certain percentage of samples. Applying stabilization can characterize computed independent components in terms of their stability that can be further used for ranking them. For example, it was demonstrated that such ranking is usually more meaningful in the case of transcriptomic data analysis compared to other methods of component ranking (e.g., by the measure of non-Gaussianity or by the explained variance) [34]. One of the first and most popular approaches to ICA stabilization is the *icasso* method, introduced by the creators of fastICA [41]. Interestingly, in the case of transcriptomics data, the most stable independent component frequently strongly correlates with the first principal component.

Lastly, in some applications of ICA (e.g., cell-type deconvolution), it is desirable to fix the orientation of the independent components. We remind that in PCA and ICA, the signs of the elements in the vectors ***a_k_*** and ***s_k_*** can be inverted simultaneously without changing the definition of the component. Some methods (such as BIODICA or DeconICA) avoid this ambiguity by assuming that the heaviest tail of the ***s_k_*** distribution should correspond to positive values, which usually gives satisfactory results. In Reference [43], each ICA component was characterized by two sets of top contributing genes, from the negative and the positive side of the metagene weight distribution. The largest such set was called a dominating module and the final orientation of the component was chosen to make the weights of the dominating module positive. In other cases, labeling of samples can be used in order to select one of the two possible signs of ***a_k_*** and ***s_k_***. In this case, the orientation was chosen based on the values of ***a_k_*** vectors. For example, in a disease study, one can require that any component would be oriented towards aggravation of the disease condition (e.g., from normal samples to more aggressive cancer stages). This approach was recently used for quantifying disease comorbidity using ICA [44].

### 2.4. Assessment and Comparison with Other Matrix Factorization Methods

In several recent studies, ICA was systematically compared with the other most used matrix factorization methods such as PCA and NMF, using large collections of cancer omics measurements. 

In Reference [38], it was tested which matrix factorization method could produce the most reproducible (i.e., generalizable) definitions of metagenes. In order to achieve this, a notion of a reciprocal best hit (RBH) graph was borrowed from evolutionary bioinformatics. Reciprocal best hit between two metagenes in two ICA decompositions of different datasets defined “orthologous” metagenes. Several criteria have been used in order to evaluate the modular structure of the RBH graphs resulting from application of various ways of applying ICA, PCA, and NMF to the transcriptomic data. In particular, the total number of RBH relations among the components, average clustering coefficient and modularity of the RBH graph, and the number and typical sizes of the identified graph communities have been assessed. The conclusion was that the stabilized version of ICA, where the non-Gaussianity of metagenes (and not metasamples) was maximized, is superior to other matrix factorization methods with respect to these measurements. 

Three major matrix factorization approaches were systematically discussed in a recent review for their ability to discover functional subsystems or tissue-type specific signals [45]. The main conclusion was that it might be advantageous to use several matrix factorizations simultaneously. The same authors further suggested using the BioBombe approach [46], where three matrix factorization methods (PCA, ICA, and NMF) and two autoencoder-based dimension reduction techniques were systematically compared based on the pancancer TCGA datasets comprising 11,069 tumoral samples. Indeed, each data decomposition method showed its own advantages with respect to different tests and tasks. For example, the ICA method outperformed other approaches when the extracted metagenes were tested for gene set coverage of specific gene set collections representing transcription factor targets, Reactome pathways, and cancer modules. Higher gene set coverage in this study meant the proportion of gene sets in a reference collection, which could be significantly associated with one of the metagenes in the decomposition.

### 2.5. Estimating the Number of Independent Components

The most important parameter in the application of any matrix factorization method is the number of components to determine. This question is less crucial in the case of PCA due to the orthogonality constraint and that computing higher-order components does not affect the definition of the lower-order ones. However, this is not the case with ICA and NMF: choosing the order of decomposition affects the definition of *all* computed components. In the case of ICA, which geometrically only rotates the PCA axes, choosing the number of independent components can rely either on the methods for determining the number of relevant principal components or it can use some features of the independent components themselves in order to determine the optimal decomposition order.

In the first case, the effective global dimensionality of the data can be determined through the standard Kaiser rule, use of broken stick distribution, Horn’s parallel analysis or estimating the conditional number of the covariance matrix [47]. One can also use more advanced methods for determining the effective data dimensionality such as the ones using concentration of measure phenomena [48] or data point cloud linear separability statistics [49]. 

However, the second case appears to be more consistent in applications, even being computationally more challenging. Thus, in Reference [24], Bayesian information criterion (BIC) was exploited to determine the optimal number of independent components in the analysis of a metabolome dataset comprising 1764 samples and 218 measured metabolites. The optimal number of components according to this estimate appeared to be quite small (eight). 

In Reference [34] stability indices of independent components were used in order to define so-called maximally stable transcriptomic dimensionality (MSTD) measure, in case of transcriptomic data. The MSTD defines an order of transcriptomic matrix decomposition such that the distribution of stability indices for independent components is not yet dominated by highly unstable ones. It was demonstrated that the independent components within the MSTD range are characterized by better reproducibility and interpretability. Based on the analysis of a large volume of cancer transcriptomic data, several observations were made. Firstly, unstable higher order components are frequently driven by very few (frequently, only one) genes. In other words, their ***s_k_*** distributions are characterized by the presence of one or few weights with exceptionally large values, separated by a significant gap from the other values. Secondly, it was shown that a certain level of *over-decomposition* of transcriptomic datasets, i.e., choosing the number of independent components several times larger than MSTD, does not drastically change the definition of most of the components within the MSTD range. At the same time, it was observed that increasing the number of independent components over the MSTD value sometimes leads to biologically meaningful splitting of the components. For example, a component within the MSTD range which was associated with the total level of immune infiltrate in tumoral microenvironment splits into three components in higher-order decompositions which can be associated with the presence of T-cells, B-cells, and macrophages [34,42].

In Reference [46], a range of decomposition orders have been tested using various criteria for several matrix factorization methods. The general conclusion was that it can be advantageous to use multiple-order decompositions if the aim is signature discovery. Just as in Reference [34], it was shown that higher-order matrix factorizations with at least 40–50 components provide more precise interpretation with respect to associating the components to the clinical information such as patient gender or to the mutation status of cancer driver genes.

### 2.6. Methods for Interpretation of Independent Components

Assigning a meaning to the extracted independent factors remains a major problem in exploiting ICA in biological research. Standard practice consists of applying various kinds of functional enrichment analyses to ***s_k_*** vectors (e.g., applying hypergeometric test or overrepresentation analysis (Webgestalt 2017) to the set of most contributing genes, or Gene Set Enrichment Analysis to the whole ranking defined by ***s_k_***), using large-scale collections of reference gene sets. The distribution of gene weights from ***s_k_*** vectors can be projected on top of genome-wide biological network reconstructions where the network edges represent different types of interactions or regulations between genes and/or proteins. This can be further used for various types of network-based analyses, leading to the determination of biological network “hotspot” areas and eliminating the need of having a reference gene set collection [50]. The ***s_k_*** vectors (resulting from the analysis of transcriptomic or methylome data) can be projected onto genome and be a subject of peak-calling analysis, which can sometimes lead to associating a component to genomic alterations [33]. 

Metasample weights ***a_k_*** are used to associate components to sample annotations such as clinical data (tumor stage, molecular classification, time label, sample processing data, etc.). Metasamples can be also associated with some clinically relevant molecular data, such as mutations in known cancer drivers for a particular cancer type. Metasamples can be also associated with known labels for molecular tumor subtype.

In parallel to rigorous statistical testing, insightful visualizations of the results of ICA application can be of great help. For example, gene weights from ***s_k_*** vector can be projected on cancer-specific biological network maps such as the Atlas of Cancer Signaling Network (ACSN) using user-friendly Google Maps-based online platforms such as NaviCell and MINERVA [51,52]. Functional enrichment analysis results of ICA metagenes can be visualized using maps representing functional redundancy between reference gene sets, such as InfoSigMap or enrichment maps [53]. 

There exist integrated solutions allowing the computation of ICA components for omics datasets and containing a built-in set of tools for their interpretation. For example, in the BIODICA package (Available online: https://github.com/LabBandSB/BIODICA), a set of tools is provided for performing hypergeometric tests of the metagenes, automated feeding of Gene Set Enrichment Analysis with ICA metagenes, projecting metagenes onto biological network maps, correlating computed metagenes with a reference database of previously annotated metagenes, associating components with categorical and numerical sample annotations, and tools for meta-analysis of ICA decompositions. 

**Figure 3:**
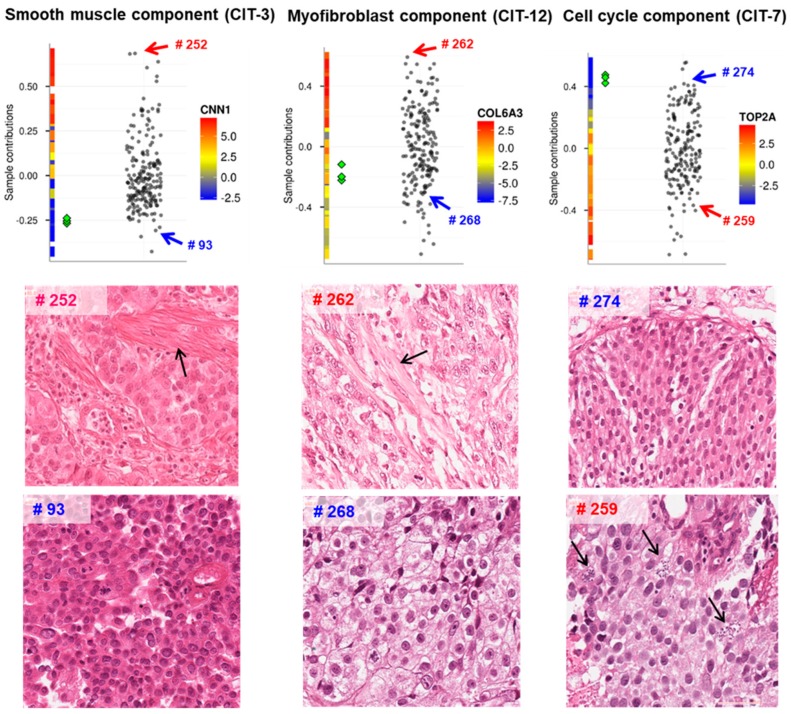
Interpretation of ICA components using histopathology imaging of bladder tumor cross-sections. Each metasample produced by ICA defined a ranking, which was used to sort the images. Visual inspection determines a clear trend in the images towards the increase of certain elements (presence of smooth muscle cells, myofibroblasts (cancer-associated fibroblasts), dividing cells). Two example images per component selected from the top and the bottom of the rankings are shown here. Green rhombuses designate normal samples. Black circles designate cells of interest: muscle cell (left), myofibroblast (middle), cells in mitosis (right). The figure is reproduced from the Supplementary Materials of Reference [33] with permission.

A particular interest represents joint analysis of omics profiles together with histopathological imaging data. A simple analysis was made in Reference [33], where the independent components computed from the transcriptomic data were used to rank the matched histopathological images according to the contribution of the corresponding tumor sample to the component. This simple approach was used in order to confirm the biological meaning of some of the components (see Figure 3). Today this approach can be further elaborated and automated by applying machine learning-based methods for extracting features from medical images and correlating them to the patterns identified from the omics data (such as ICA metagenes), which can lead to getting new insights into cancer biology [54].

## 3. Applications of ICA in Cancer Research

### 3.1. Applications to Data Preprocessing, Classification, Dimensionality Reduction, and Clustering

In multiple studies, ICA was shown to be efficient in disentangling biological and technical factors affecting molecular profiles. This supports the idea to use ICA as a powerful data preprocessing and/or feature engineering method for further application of machine learning methods. The general approach is to apply ICA as an unsupervised machine learning method, to decide on the biological meaning or the technical origin of individual components and then focus on a subset of them containing the relevant signal. This can be achieved either by directly using the relevant subset of components as features or by constructing a modified matrix of molecular measurements which would be free of the influence of those components which are identified as non-relevant or of technical origin.

Frequently, each one-dimensional ***s_k_*** distribution is analyzed for determining a set of the most contributing genes (e.g., characterized by the most extreme absolute values in ***s_k_***). The simplest idea is to select the variables (e.g., genes) bypassing the threshold in *p* standard deviations, with some choice for *p* (typically, *p* ≥ 3). A combined set of the most contributing to different ICs genes can be used to define a subset of data for further analysis.

Interestingly, ICA decomposition can be used to identify and disregard technical biases among omics datasets produced by different platforms. For example, in the study of 198 bladder cancers in Reference [33], one of the most stable components was found to be associated with a complex time-dependent batch effect. The nature of this batch was not known in advance and was only discovered by correlating the corresponding ***a_k_*** vector to the dates of sample preparation. Another component frequently identified in the analysis of transcriptomic data is related to GC-content, which might reflect the influence of GC-content on the RNA amplification step common for both microarray-based and sequencing-based methodologies. In Reference [39], a small dataset of three primary melanoma tumors and two matched controls, characterized at the level of transcriptome and miRNA, were merged together with a large reference melanoma dataset from the Cancer Genome Atlas. The ICA decomposition was performed for the merged transcriptomic and miRNA data separately. For both molecular data types, it was possible to identify those independent components capturing technical differences among platforms while focusing the analysis on biologically meaningful factors whose quantification was comparable among platforms.

Interestingly, ICA-based analysis sometimes can lead to identification of the factors whose origin is intermediate between technical and biological. For example, in Reference [33] one of the factors reproducible in several bladder cancer datasets was strongly associated with the surgery type (transurethral resection of the bladder tumor versus cystectomy) and at the same time was enriched with early response genes. This suggests that different ways of tissue processing might leave characteristic patterns in the transcriptome which can be discovered using ICA.

Some components identified through ICA could describe various cell populations present in the sample in addition to cells of direct interest. Typically, this was the case for the stroma-related signals in the ICA-based analysis of tumor bulk samples (see Figure 2). ICA can efficiently deconvolute the contribution from the cells of different types to the bulk transcriptome, which allows studying the properties of tumor cells more directly. In the aforementioned study of bladder cancer, decomposition of bulk tumors into 20 components allowed for the clear distinction of the signals reflecting the presence of immune cells (with the main signal coming from the multiple types of lymphocytes, adipocytes, fibroblasts [33]. 

Another frequently employed idea is to use the results of ICA decomposition in order to define a set of variables for further application of various machine learning methods. Zhang et al. [55] were among the first who applied ICA as a data-preprocessing step for classification of cancer patients. They used ICA independently on normal and cancer datasets and identified top gene markers able to discriminate between these conditions. Their approach was quite indirect but showed the ability of ICA to prioritize genes. In a study by Huang et al. [56], ICA was followed by a penalized discriminant method, and the authors showed high accuracy of ICA-based approach on several datasets. In both mentioned papers, the authors segregated cancer and normal tissues, which is now considered a trivial task, taking into account the large effect of cancer on cell transcriptome. Later, Zheng et al. [57] proposed a consensus ICA, robust to initial estimations. They showed the applicability of the approach on three datasets, in two of which they classified subtypes of tumors. Support vector machine (SVM) was used to predict classes based on the metasamples, and the authors needed to perform preliminary feature selection to improve their classification accuracy.

Recently ICA was used to engineer features for further use in cancer-related classification tasks, using naïve Bayes classifier [58]. In Reference [59], ICA was used as a data pre-processing step in order to improve the clustering of temporal RNA-Seq data. It was suggested to use ICA in combination with wavelet-based data transformation in order to engineer transcriptomic features at “multiple resolution” [60] and use them to improve tumor classification and biomarker discovery. In Reference [22], it was shown that a set of 139 features built by systematically applying ICA to a large cohort of transcriptomic profiles, can be directly used in machine learning for classification tasks and have advantageous characteristics in small sample studies, compared to the classical differential expression-based feature selection. It was noticed also that using ICA-based features reduced to some extent the batch effects when clustering the transcriptomic data. 

Any matrix factorization method can be used for dimensionality reduction. The specifics of ICA are in that it is usually performed in an already reduced space and only defines a new coordinate basis in the principal linear manifold. Therefore, ICA itself does not reduce the data dimension more that it is done by PCA. Nevertheless, it is a frequent practice to consider the coordinate basis defined by few independent components as a subspace to further application of various data analyses. For example, this approach is used for a standard pipeline of single cell RNA-Seq data analysis [61]. Similar notice can be made with respect to using ICA as a data visualization tool. Selecting a couple of independent components with clearly identified biological meaning can lead to a biologically meaningful 2D data display. For example, in Reference [62], visualizing a single cell dataset in the plane of two independent components associated with proliferative genes clearly revealed the 2D dynamics of tumor cell progression through the cell cycle. The difference with PCA-based data visualization is that, in the case of ICA, there exists no principal pair of components (such as PC1 and PC2) which can be considered as the most representative for visualizing the multi-dimensional distances. This remark should be taken with care since, frequently, the first PCs are affected by technical artifacts and are to be neglected in further analysis. In the case of ICA, any pair of ICs in no particular order can be used for data visualization taking into account their tentative interpretation. Examples of contrasting PCA and ICA approaches for data visualization can be found in References [37].

### 3.2. ICA for Unraveling Functional Subsystems of a Living Cell or a Cell Ecosystem

One of the strongest motivations behind applications of ICA to omics data is in that it can help identifying functional subsystems (or functional modules and complex biological processes) which are the building blocks determining response to perturbation of a tumoral cell or a whole cellular ecosystem such as tumor microenvironment (TME) composed of different cell types. The underlying principle is that genes or proteins do not react to an external stimulus individually but always integrate into a (sub-)system with more or less defined limits. Importantly, it is biologically feasible to assume the phenomenon of plurifunctionality, i.e., potential participation of an elementary entity (such as gene or protein) into several functional subsystems. 

The composition of a functional subsystem is defined by a matrix factorization method in the form of the ***s_k_*** vector (weights associated with the omics variables) or metagene. The level of activation (or inhibition) of an identified functional subsystem ***s_k_*** can be read in the corresponding metasample vector ***a_k_***. The same is relevant for an independent component associated with a technical factor intensity.

If no explicit sparsity constraint is imposed when computing the vectors, then each omics variable (gene, protein) has a non-zero contribution (estimated by its weight in ***s_k_***) to the definition of the subsystem, which can be positive or negative. However, those variables having close to zero weights can be neglected from the subsystem definition. An important characteristic of a metagene is the set of the most contributing genes (see discussion in the previous section). The most contributing genes are useful to characterize the functional subsystem and to identify if this subsystem corresponds to an existing known one. After determination of the sets of the most contributing genes per each metagene (functional subsystem), one can check if a gene is associated with the subsystem exclusively or contributes to several ones. This analysis can be used to identify potential coupling between the subsystems and their concrete mechanisms (see further discussion). Sometimes it is convenient to distinguish two gene sets per metagene, having the largest and the smallest set of weights, from the positive and the negative sides of the ***s_k_*** distribution. 

We can distinguish two types of functional subsystem response. One is due to the mechanistic downstream effect of a stimulus, i.e., through an induction of a transcription factor downstream of a signaling pathway. Another type is a more systemic one, indirect and related to a longer time scale, caused by an adaptation of the whole system to the presence of potentially harmful factors (such as hypoxia or active immune response) [63,64]. If a studied system’s response (e.g., a tumor cell) is measured in a sufficiently variable number of conditions or perturbation types, one can hope to identify the composition of the most relevant/responsive functional subsystems by applying an appropriate machine learning methodology.

Identification of functional subsystems (modules) from cancer omics data was first historically approached with hierarchical clustering of genes [65]. Matrix factorization in this sense seems to be a more suitable mathematical formalism since it naturally allows taking into account the gene plurifunctionality. This is a simple consequence of a gene that can significantly contribute (i.e., be in the list of the most contributing genes) to the definition of several functional subsystems. ICA is a powerful approach here, because the requirement of maximally possible statistical independence seems to be well suited for the task of subsystem identification. Even if the activity of a pair of functional subsystems is correlated in the most of observed conditions, ICA can still distinguish them based on a smaller number of conditions when they de-synchronize (see a discussion of this aspect in the methodological part of the review). This ability of ICA is also powerful in disentangling the technical biases from biologically relevant signals (as discussed in Section 3.1), making the identification of the functional subsystems less prone to technical biases. Last but not least, ICA allows taking into account the case when the activation of a functional subsystem is connected to inhibition of some of the genes or proteins. One of the simple examples of such a situation is when a transcription factor has a role of an activator for some genes and an inhibitor for other genes. 

Functional subsystems identified by ICA can reveal an important coupling of several known biological mechanisms and relate it to the biological phenotype such as cancer patient outcome. In the case of breast cancer, this phenomenon was described in Reference [27] through so-called ICA-based association networks. 

One important characteristics of the weight distribution composing ***a_k_*** is the unimodal or bi- or multi-modal character of the distribution. In the case of well-defined bimodality of a metasample, one can stratify the distribution of samples into two groups, with respect to the nature of the functional subsystem identified. A typical example of this kind is the identification of the functional subsystem of proliferation in single-cell RNA-Seq data, where the corresponding metasamples frequently have two modes, corresponding to proliferative and non-proliferative cell states.

Functional subsystems have been systematically identified using ICA from a large cohort of transcriptomic profiles in Reference [43], where 298 Gene Expression Omnibus (GEO) datasets profiling 9395 human samples (from various conditions including cancer samples) were used to identify 423 “fundamental components of human biology”. As an example of their use, the authors characterized the molecular mechanisms of parthenolide anti-cancer drug action. Recently, similar large-scale analysis has been applied to a larger dataset, containing 2753 datasets and 97,049 samples [22]. Compared to the earlier study, the authors improved the methodology in order to avoid redundant and correlated transcriptional component definitions, applying Horn’s parallel analysis in order to select the optimal number of components and systematically evaluating the components’ reproducibility after resampling. This analysis resulted in defining 139 reproducible and informative transcriptional modules whose value for the downstream analysis was explicitly demonstrated.

**Figure 4:**
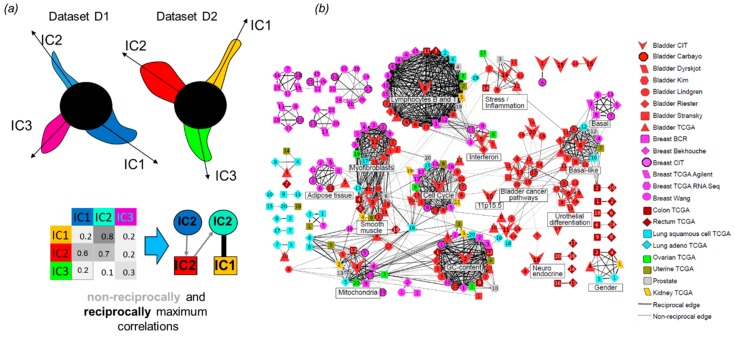
Use of ICA components in meta-analysis of multiple omics datasets. (**a**) Pairwise comparison of two sets of ICA metagenes led to an asymmetric correlation matrix (same as in Figure 2c) which can be converted to a graph using some threshold and selecting the *maximal* correlations. If two components are maximally correlated with each other, then such a correlation defines reciprocal best hit (RBH). (**b**) Graph of maximal correlations (reciprocal and not) exceeding certain threshold among components computed for 22 cancer transcriptomic dataset. Each node is a component, and an edge denotes a correlation. Color reflects the cancer type (e.g., red is bladder cancer). Communities in this graph define highly reproducible cancer type-specific and universal latent factors The figure is reproduced with permission from Reference [33].

Identification of the functional subsystems and distinguishing them from potentially technology driven factors can be strongly improved by the application of ICA analysis to multiple similar datasets independently (without merging them). In this scenario, the ICA results from several datasets were compared with each other in terms of the correlation or other suitable similarity measure among metagenes (Figure 4). In the case of cancer, one of the first applications of this approach was done in Reference [27] for 800 breast cancer samples from four independently profiled cohorts with a conclusion that independent components matched well the underlying cancer mechanisms. This type of meta-analysis was further upscaled in Reference [33], where 22 non-redundant cancer transcriptomic datasets were analyzed. Some of the datasets were related to the same cancer type, i.e., eight of them were collecting samples of bladder cancer and six were from breast cancer. Because the datasets used in this study were produced using different technological platforms, this analysis identified the technical biases captured by individual components in specific datasets and not reproduced among others. It also distinguished cancer type-specific functional subsystems (such as differentiation program of urothelial tissue) and generic and potential pancancer-wise important functional subsystems (such as the transcriptional program of proliferation or oxphos). Interestingly, one of the bladder cancer-specific components associated with differentiation of urothelial tissue was also associated with amplification of a genomic region, containing a particular transcription factor (PPARG). This led to a conclusion about the role of PPARG in differentiated bladder tumors which was validated experimentally. In Reference [38], 14 non-redundant colon cancer transcriptomic datasets were analyzed by ICA, and the resulting ***s_k_*** vectors were matched with each other through correlation in order to reveal the functional modules implicated in colon cancer tumor cells’ and the variability of tumoral microenvironment.

In the case of a very good match between ICA-based metagene definitions from several independent datasets, one can define a consensus metagene definition from the meta-analysis (a meta-metagene). An exemplary set of such reference metagenes was built in Reference [33] and used in other studies to facilitate the interpretation of the ICA results. This set included (a) ICA-derived and universal for many cancer types of proliferation-, oxphos-, immune infiltration-, interferon signaling-associated metagenes; (b) consensus metagenes associated with the presence of non-tumor cells of several types in TME; and (c) bladder cancer-specific transcriptional modules (such as differentiation program of urothelial tissue). A comprehensive catalogue of ICs identified in the pan-cancer TCGA dataset containing 32 cancer types was produced in Reference [34]. It appears to be a useful effort to extend the collection of reference consensus metagenes, since they seem to be highly generalizable (reproducible in independent datasets) [22,38].

### 3.3. Applications to Unsupervised Cell Type Deconvolution

In cancer biology, bulk omics data (especially transcriptome and methylome) represent heterogeneous samples such as peripheral blood mononuclear cell (PBMC) and tumor biopsies, in which the expression profiles of distinct cell types are mixed in each sample at a priori unknown proportions. The tumor microenvironment is composed of many different cells including a plethora of immune cells, stromal cells, and blood and lymphatic vessels [66]. The quantities and the nature of the TME compartments change with the cancer type and cancer stage. Recent works showed that immune cells could influence tumor cells in different ways and that the immune therapies take advantage of the protective function of the immune system and aim to activate patients’ immune defense. 

**Figure 5:**
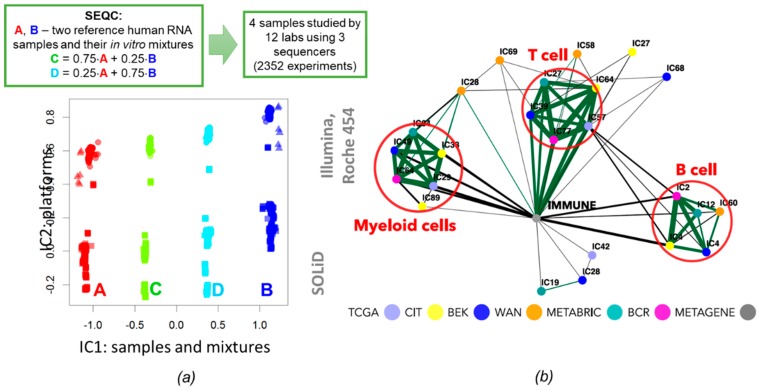
Examples of utility of ICA for unsupervised deconvolution of cell types. (**a**) Application of ICA to the Sequencing Quality Control consortium (SEQC) dataset [76] containing measurements of two references transcriptomic profiles of cell lines and their mixtures at known proportions. The first two ICs identify the types and the effect of the platform. (**b**) Correlation graph among selected components from ICA applied to six non-redundant breast cancer transcriptomic datasets. Three cliques formed in the graph correspond to major immune cell types. The thickness of the edges reflects the absolute correlation value. “Immune” meta-metagene was defined in Reference [33] as the one associated with the presence of immune infiltrate in a tumor. This figure was reproduced with permission from Reference [42].

Therefore, one of the major challenges for computational analysis of bulk samples is evaluating the proportions and the properties of individual cell types composing the sample, frequently called deconvolution problem in this context [32]. In general terms, deconvolution stands for unmixing a mixture, which makes it close to the blind source separation methods, including ICA (Figure 5a).

Deconvolution of cancer bulk transcriptomes gained a lot of popularity in the last several years due to the abundance of data sources. Several methods were proposed to estimate the abundance of immune infiltration in cancers at different levels of granularity [67,68,69,70] using a pre-defined set of genes, usually generated from pure blood cell population gene expression data [67,68], from single-cell RNA-Seq measurements [70] or mixed [69]. They were proven to correctly estimate the cell-type abundance in silico simulated datasets, in vitro cell mixtures, and blood or PBMC transcriptomes coupled with fluorescence-activated cell sorting (FACS) estimations. However, it remains unclear how many cell types or cell states can be quantified from bulk transcriptomes as each tool comes with own definition of cell-types (e.g., T-cell) and subtypes (e.g., CD4-activated T-cell) or cell states (e.g., cytotoxic T-cell).

In response to this problem, reference-free (also called unsupervised) approaches propose a more data-driven way of performing the deconvolution. This group of approaches is able to discover the cell types and their markers as well as approximate profiles of those cell types (perform “complete deconvolution”). Different types of matrix factorization are suitable for solving this problem. Even though these deconvolution methods are called reference-free, known reference profiles are used to interpret and select the cell type-related components. Different possible benefits of reference-free approaches can be listed as (a) flexibility—discovering the context-dependent cell-type markers, (b) discovery of new cell types or cell types that are specific to a certain context, (c) determining deconvoluted profiles of cell types that can be used to remove the immune-related signal or to better understand the cell type features, and (d) ability to characterize biological processes (such as cell cycle activity) simultaneously with the cell types.

The reference-free approaches were already applied for deconvolution of cell types in blood using semi-supervised NMF [71]. They were used to study brain [72], tumoral single cells [73], and cell-cycle in yeast [74].

In Reference [42] icasso-stabilized fastICA was applied to a set of six large breast cancer patient cohorts profiled for gene expression. It was demonstrated that the immune-related factors, especially the signal of T-cell, B-cell, and macrophages were highly reproducible in independent datasets (Figure 5b). In Reference [75], the DeconICA R package (Available online: https://github.com/UrszulaCzerwinska/DeconICA) was developed with the objective to apply ICA to the task of cell type deconvolution. It was shown that ICA is able to efficiently estimate the cell type proportions with better accuracy than leading supervised algorithms even though it can identify less cell sub-types than most of the published solutions. It suggests that ICA-based deconvolution is less prone to overfitting and enables discovery and quantification of strong and stable signals (not necessary the most abundant but rather the most specific). DeconICA was applied to a big corpus of data containing more than 100 transcriptomic datasets composed of over 28,000 samples of 40 tumor types generated by different technologies and processed independently. In addition, the ICA-derived metagenes were used as context-specific signatures in order to study the characteristics of immune cells in different tumor types. The analysis revealed a large diversity and plasticity of immune cells dependent and independent on tumor type. Some conclusions of the study can be helpful in the identification of new drug targets or biomarkers for immunotherapy of cancer. 

Cell-type composition can be also computed from DNA methylation data. In the EWAS (Epigenome Wide Association Studies), the variation origination from cell types is considered as an important confounding factor that should be removed before comparing cases and controls and defining Differentially Methylated Positions (DMPs). For example, in Reference [77] ten tools for epigenome deconvolution were reviewed. The authors described six reference-free methods, three regression-based, and one semi-supervised. Some of these methods use approaches close to ICA, such as independent surrogate variable analysis (ISVA) [78], where the goal is to adjust the data for any type of confounder (be it cell-type composition or not). Clear superiority of ICA over PCA in methylome deconvolution has not been yet demonstrated. Most of the existing tools for unsupervised methylome deconvolution assume cell composition as the most contributing to the methylome variability. According to Reference [78], this assumption was not proven to hold true in solid tissues, normal or pathological. It appears to be interesting to test different approaches to ICA coupled with improved reference profiles to check if it cannot open new perspectives in methylome deconvolution.

### 3.4. ICA Applications to Single-Cell Omics Data Analysis

Statistical properties of ICA seem to be very attractive to justify its application to the emerging wealth of single-cell omics data profiles. ICA can serve here to improve the data analysis regarding dimensionality reduction, removing technical biases, integrating datasets. ICA also looks promising and represents an alternative to the standard dimensionality reduction followed by clustering methodology for identifying cell types or states, suggesting a more continuous way of considering them, with a possibility of existence of intermediate or mixed cell populations.

**Figure 6:**
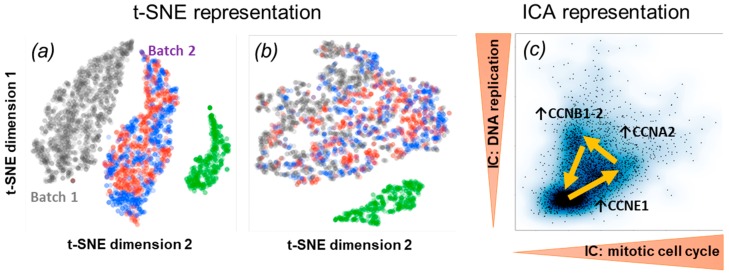
Application of ICA in single cell data analysis of tumors (study of glioblastoma from Reference [79]). (**a**) t-distributed stochastic neighbor embedding (t-SNE) visualization of the data reveals a strong batch effect. Grey and red/blue dots represent cells from the same cell line, analyzed in two batches (batch 1—grey dots, batch 2—red and blue cells). The green dots show a cell population from a different cell line added to the dataset for the reason of comparison. (**b**) t-SNE visualization of the data after eliminating signals contained in one IC associated with batch effect. (**c**) In ICA decomposition of single cell scRNA-Seq data from cancer studies, usually there exist two components associated with phases of the cell cycle (G1/S, DNA replication, and G2/M, mitosis). Here the loadings of such two components are visualized. Black arrows show the regions when the labeled genes are highly expressed. Yellow arrows show assumed direction of the progression through the cell cycle.

Similar to bulk RNA-seq, technical biases and batch effects are limiting factors for single-cell RNA-Seq and should be either removed or taken into account. One example of ICA application to normalize batch effect was recently reported by Dirkse et al. [79]. The authors observed a strong difference between the original patient-derived cell line and its two subpopulations measured in the second batch (all cells undergo the same protocol of cell growth and sorting, so biological differences were excluded). The difference among batches was comparable to the difference among different cell lines. The ICA identified and isolated the batch effect in one of the components. By removing this component and recalculating expression matrix, the authors corrected for this batch effect (see Figure 6a,b). A similar approach was exploited in Reference [80] in order to pre-process the single cell data following the trans-differentiation process of murine pre-B cells into macrophages and their reprogramming into induced pluripotent stem cells. In this study, 15 out of 35 independent components were considered to be connected with technical artifacts such as sample batch effects and cell position in the plate and filtered out from the downstream analysis.

The ICA-based dimensionality reduction is a standard step in the most popular packages for analyzing single cell RNA-Seq data. In MONOCLE [61,81], ICA is optionally used for the initial step of dimensionality reduction to 2D, before inferring cellular trajectories. For example, this option was used in order to derive the cellular trajectory of individual MCF-7 breast cancer cells after stimulating them with estrogen [82]. It is also part of the popular toolbox Seurat [83] as one of the standard choices for dimensionality reduction, data visualization, and feature selection. ICA can be exploited, together with other low-dimensional projections, in various recently developed packages for biologically meaningful single-cell data visualization [84].

In Reference [85], ICA was applied in order to define subtypes of the immune-related cells present in the TME of melanoma (with original data from Reference [86]) and relate them to the mechanisms of innate immune response. ICA was used to define the continuous spectrum of differentiation in hematopoietic cells from scRNA-Seq data in Reference [87]; several latent factors were associated to the underlying biological mechanisms of differentiation. In Reference [80] three independent components computed for a scRNA-Seq dataset were matched with transcriptional programs specific to B-cells, macrophages, and monocytes and used to provide an interpreTable 3D data visualization. Interestingly, in order to establish the biological origin of these components, they were correlated to the ICA decomposition of the transcriptomic atlas of murine cell types from which 120 independent components were extracted.

ICA served as a principal machine learning method for discovering functional subsystems involved in the response of Ewing sarcoma cells to the induction of the chimeric oncogenic transcription factor EWSR-FLI1 [62]. In this case, ICA was applied to the temporally resolved single cell RNA-Seq dataset and revealed the existence of few tens of transcriptional programs activated or inhibited after the controlled induction of the oncogene. Quite remarkably, one of the independent components was clearly associated with the functional subsystem composed of the direct targets of EWSR-FLI1, and it was distinguished from its indirect downstream effects such as cell cycle induction (see Figure 6c). Other functional subsystems reacting to the variations of the experimental conditions such as hypoxia or regulation of glucogenesis, were recapitulated in individual ICs. Identification of the functional subsystems from the cell line experiments were further used in order to characterize the patient-derived xenografts (PDXs) of Ewing sarcoma, at single cell level.

In principle, ICA is the methodology able to exploit strong non-Gaussianity in the multidimensional distributions formed by single cells in the space of omics profiles. However, in order to optimally use this potential, one probably needs to identify the most suitable non-linearity functions, for each particular type of single cell measurements, and take into account the nature of the multivariate distribution of points in data space. Recently, a matrix factorization-based method ZINB-WaVE was adapted to the single cell RNA-Seq measurements, using the model of zero-inflated negative binomial distribution (ZINB) [88]. In principle, ICA approach can be applied on top of ZINB-WaVE instead of PCA; however, this approach needs to be tested in practice.

### 3.5. Multi-Omics ICA Applications in Cancer Research

The majority of published works on applying ICA in cancer research deals with transcriptomic data. This is connected in part to the relatively high abundance of such data type from collections of bulk tumors, and in part to the availability of bioinformatics tools helping to interpret the obtained components (such as Gene Set Enrichment Analysis). Yet another aspect is that transcriptomic data are better connected so far to the clinical questions such as defining molecular subtypes of tumors. 

However, applying ICA should not be limited to only one level of omics profiling, and there is a lot of potential in applying it to several levels of molecular description. The multi-level datasets become increasingly available in the cancer biology. The levels of molecular description can be gene copy number profiles, binary mutation profiles, measured mutational signatures, measured total expression of genes or spliced mRNA isoforms or non-coding genes such as microRNAs, DNA methylation or histone mark modification profiling, protein or protein phosphoforms relative abundances or some other less frequently used omics types. Identification of functional subsystems can be facilitated through the use of several data types, since the adaptation process is frequently expected to span several levels. As a good example of such a multi-omics dataset, one can cite recent work on comprehensive characterization of medulloblastoma [89].

Ideally, several levels of omics profiling should be collected for the same and sufficiently large set of samples. Independent components can be then computed for each data type separately and then the identified components can be compared by computing correlations between the corresponding ***a_k_*** vectors (metasamples). Such an approach was recently applied in Reference [39] to a set of melanoma bulk samples, profiled at the level of transcriptome and microRNA expression. Similarly, in a recent study [90], 77 breast and 84 ovarian cancer samples, profiled simultaneously at transcriptome and proteome level, were analyzed using stabilized ICA, followed by integrating the discovered associations with clinical data and molecular pathways.

An alternative and somewhat more powerful idea consists in stacking several matrices corresponding to the different levels of omics profiling into a tensor (multi-dimensional array), in order to apply the tensorial version of ICA. In this case, ICA will be able to learn and jointly optimize the signals which can involve variables from several levels of molecular description. This requires making at least two dimensions of the data common, while the third matrix dimension indicates the data type. Typically, all molecular measurements are mapped onto the genes through application of procedures that can be non-trivial (e.g., in the case of Chip-Seq experiments). 

The resulting three-dimensional measurement tensor *X_ijk_* has dimensions “number of samples × number of genes × number of data types”. For example, *Xi* = 4, *j* = 5, *k* = 2 element in the tensor can indicate DNA methylation level of the promoter of the gene 5 in the sample 4. *Xi* = 4, *j* = 5, *k* = 1 could indicate expression of the same gene in the same sample. In the case of tensor factorization, the resulting components represent matrices rather than vectors having dimensions “number of genes versus number of data types” (for metagenes) and “number of data types versus number of samples” (for metasamples). The existence of correlations among different data types within the same matrix-component indicates coupling among several levels of molecular descriptions captured by tensorial ICA.

Tensorial ICA was recently applied in Reference [91] to colon cancer dataset from The Cancer Genome Atlas (TCGA) composed of a matched subset of copy-number variation (CNV), DNAm, and RNA-seq data. A specific implementation of tensorial ICA called tWFOBI, standing for tensorial fourth-order blind identification, accompanied by a tensorial version of whitening (W), using tensor PCA, was used to compute 37 independent components. Most of these components can be associated with the differences between normal and cancer samples, while only four components capturing correlations between CNV and gene expression, and one among them was also characterized by concomitant correlation among all three data types. Of note, the tWFOBI method showed several orders of magnitude better computational performance compared to the state-of-the-art methods developed for multi-level omics data integration (such as iCluster).

Applications of ICA to data types other than transcriptomic or to several data types simultaneously remain limited; however, first applications of this approach in cancer biology are rather promising [91,92]. Multiple issues still remain to be solved for how to define the best practice of ICA application to, for example, DNA methylation profiles and how to interpret the obtained results. For example, in Reference [93], a “spatio-temporal” version of ICA was suggested in order to take into account certain specificity of DNA methylation profiles such as a high level of correlation among probes located close in the genome. Also, in the case of methylation data, ICA should be carefully benchmarked with other machine-learning methods exploiting the non-Gaussian nature of signals [94].

### 3.6. Correlations and Interactions among Functional Subsystems Defined by ICA

Functional subsystems identified through ICA and fixed in the form of metagenes can be studied for their statistical relationships within a dataset, among datasets of the same kind, or among datasets that are not closely related in terms of the nature of the biological samples profiled. 

In the latter case, one can use ICA for studying disease–disease relationships. An example of such a relation is the phenomenon of inverse comorbidity between cancer and some other diseases, in terms of the anti-correlated activation pattern of the common functional subsystems. For example, the ICA method was exploited in Reference [95] in order to identify inversely associated transcriptional modules common in breast cancer and Alzheimer’s disease. In a more extensive study [44], 17 transcriptomic datasets (11 collected for the post-mortal brain samples of patients suffering from Alzheimer’s disease and six collected for the lung cancer samples) have been analyzed using ICA. The notion of reciprocal best hit (RBH, see the methodological section of this review) was used in order to match the ICA components and define their communities. In order to detect the anti-correlation patterns among the matched components, a specific method was developed to assign an orientation of the components and, hence, the weight signs in the metagene, based on the analysis of the subset of normal control samples, in the ***a_k_*** vectors. This analysis confirmed previously identified comorbidity patterns based on the analysis of individual gene expression profiles (related to the role of immune system and mitochondrial metabolism) and suggested new molecular mechanisms of comorbidity between lung cancer and Alzheimer’s disease such as estrogen receptor signaling pathway or the involvement of cadherins. 

Another possibility for exploiting the ICA-based definitions of modules is to study the phenomenon of functional subsystem integration as a result of adaptation to stress or harmful conditions [63]. It was shown in many studies that the correlations among the activation patterns of different functional subsystems can be more informative than the patterns themselves [96]. ICA can deconvolute even strongly correlated signals (see Figure 2A and the Section 2.2 of this review). Also, it computes components which are as mutually independent as possible, but the level of dependence can be different even for subsets of samples within a single dataset. For example, one can expect that in the normal subset of samples, some of the functional subsystems will be less coupled with each other than in stressful conditions caused by more aggressive stages of tumorigenesis. This coupling can be caused by, for example, the shortage of essential metabolic resources making them a common limiting factor for multiple functional subsystems. If the level of mutual information between two signals increases above the ability of ICA to discriminate components, then these signals will be captured by one independent component. This phenomenon of *independent component splitting and merging* might depend on the order of ICA decomposition (and it was empirically studied in Reference [34]), on the specific biases in the composition of samples, or on the number of samples. 

The theoretical principles of functional subsystems integration have been developed [63,64]. However, it remains an interesting problem to verify and apply them to the concrete modules identifiable from the (multi-)-omics profiles. Independent Component Analysis represents an interesting option for achieving this objective.

## 4. Discussion

In recent decades, independent component analysis has become a standard tool for the analysis of tables of omics measurements in cancer biology. In certain applications, it was shown to have advantages, especially in terms of reproducibility or generalizability and biological interpretability, compared to other popular matrix factorization methods. Despite ICA being shown to be a useful tool, it seems to be under-appreciated partly due to the fact of historical reasons and partly due to the presence of existing confusions in the underlying assumptions and/or interpretation of the resulting matrix decompositions. For example, it is frequently commented that biological processes are not perfectly independent and that they are expected to be correlated in some conditions. Even though this is true, ICA can distinguish signals coupled to some extent by making the corresponding components as independent as possible. 

In this review, we made a comprehensive effort to mention most of the recent studies in cancer research where ICA served as the essential data analysis tool. We classified them into several common topics: data preprocessing, data dimension reduction and visualization, identification of functional subsystems and their correlations or interactions, deconvolution of cell types, data integration and meta-analysis. 

We also reviewed the methodological works aimed at defining the best practices of applying ICA to concrete types of omics data. Compared to the early times of applying ICA to omics datasets, today there exists a variety of implementations and improved methodologies allowing us to use the valuable idea behind ICA (exploiting the concept of statistical independence and use of higher moments of multivariate data distributions) in the best possible way. Certain progress has been made in clarifying such important questions such as determining the optimal number of components to retain or establishing the biological significance of the extracted components.

Most of the existing applications of ICA have been done so far for transcriptomic data even if the interpretation of the components used other types of molecular data (such as mutations, copy number alterations). Since recently, ICA started to be applied to other types of omics profiles, including methylation profiles and proteomics datasets. It seems interesting to determine, using ICA, independent sources of variance in newly emerging omics data types, such as systematic Chip-Seq datasets mapping the state of histone modifications or mutational signature profiles. More experience, standardization and assessment are required to use ICA in the most optimal way for the analysis of single-cell and multi-omics datasets. Moreover, in this review we did not even mention other fields of ICA application in cancer biology, including the analysis of imaging data (e.g., [97]), clinical records, and other non-omics data types, for which the ICA data model might be of interest.

Matrix factorization represents an alternative approach to the standard clustering methods, being more flexible in terms of taking into account gene plurifunctionality and ability for unsupervised deconvolution of factors whose activity can be correlated. It is worth noticing that some ICA algorithms (such as fastICA) are computationally performant when properly implemented and potentially able to deal with large amounts of molecular measurements. In this sense, ICA remains competitive vis-a-vis many other approaches (e.g., based on likelihood maximization or representation of the data in the form of multilayered networks).

We believe there are interesting directions to further explore and more deeply use the concepts behind ICA in the context of cancer biology data analyses. It would be interesting to reconsider the roots of independent component analysis into artificial neural network methodology, suggesting novel scalable autoencoding-based techniques in order to solve the problem of blind source separation adapted to the nature of the biological data. Assessing the value of supervised learning of the features extracted by ICA from omics and other data types and comparing them to “hand-crafted” or convolutional neural network-based features can lead to designing performant hybrid learning approaches, as in Reference [98]. It appears promising to take advantage of the wealth of recently emerged formalized knowledge on biological mechanisms of cancer and develop methods to inject this knowledge into the component learning process. The biological factors or functional subsystems in cells or cellular ecosystems are organized in complex hierarchies, and we need new approaches to explicitly take this into account, in order to improve the subsystems identifiably.

To conclude, as a team of authors all having extensive experience in applying independent component analysis as a tool in computational cancer biology, we advocate for its wider use in making sense of the growing amount of omics data in this and other fields.

## Abbreviations

ICA	Independent Component Analysis
PCA	Principal Component Analysis
NMF	Non-Negative Matrix Factorization
MSTD	Maximally Stable Transcriptomic Dimension
fMRI	functional Magnetic Resonance Imaging
TCGA	The Cancer Genome Atlas
BIC	Bayesian Information Criterion
FOBI	Fourth-Order Blind Identification
SEQC	Sequencing Quality Control consortium
t-SNE	t-Distributed Stochastic Neighbor Embedding
